# Inequality in remote learning quality during COVID-19: student perspectives and mitigating factors

**DOI:** 10.1186/s40536-022-00143-7

**Published:** 2022-12-26

**Authors:** Alec I. Kennedy, Ana María Mejía-Rodríguez, Andrés Strello

**Affiliations:** International Association for the Evaluation of Educational Achievement (IEA), Überseering 27, 22297 Hamburg, Germany

**Keywords:** Education during pandemic, Remote learning, Digital divide, Teaching quality, Student engagement, Inequality

## Abstract

**Background:**

Remote learning, or synchronous or asynchronous instruction provided to students outside the classroom, was a common strategy used by schools to ensure learning continuity for their students when many school buildings were forced to shut down due to the COVID-19 pandemic. Differences in technology infrastructures, digital competencies of students and teachers, and home supports for learning likely led to inequalities in the way remote learning reached and was perceived by students. This study seeks to understand how student perspectives on remote learning varied across and within several countries.

**Methods:**

Building off a conceptual framework developed to understand remote learning success and using data from the Responses to Education Disruption Survey (REDS) student questionnaire from seven countries, we construct measures of student perceptions of three essential components of successful remote learning: *Access to Suitable Technology*, *Effective Teachers*, and *Engaged Students*. We then compare values on these scales across and within countries to identify inequalities in remote learning quality during school closures. We also investigate the extent to which schools implemented supports for remote learning across countries.

**Results:**

We find evidence of across country variation in remote learning quality with certain countries having much lower values on our remote learning quality scales compared to other countries in our sample. Furthermore, we identify within-country inequalities in access to and confidence in using technology with low-SES students, girls, and those living in rural areas having lower values on these measures. Furthermore, we find some evidence of within-country inequalities in student engagement across socioeconomic groups. In contrast, we do not find as many inequalities in our measures of effective teachers. In most countries, schools provided several supports to improve remote learning.

**Conclusions:**

While inequalities in remote learning experiences were anticipated and confirmed by our results, we find it promising that, in some countries, inequalities in access to and confidence in using technology as well as student engagement did not extend to inequalities in perceptions of teacher effectiveness and support. Schools’ efforts to support remote learning, regardless of student background, should be seen as a positive and illustrate their resilience in the face of many challenges.

## Introduction

In response to concerns over the COVID-19 pandemic, educational systems around the globe made the decision to close schools to halt the spread of the coronavirus. According to UNESCO (2020), this decision impacted more than 90% of students globally. As schools were no longer able to offer face-to-face instruction, educational leaders implemented remote learning strategies to maintain learning continuity for their students. Remote learning can be defined as

Synchronous or asynchronous instruction provided in a place outside the classroom. Synchronous learning means that students are connected to learning experiences where a teachers’ immediate feedback is possible. Asynchronous or self-directed learning means that students can learn at their own pace and chosen time. Remote learning takes an array of forms ranging from paper-based take-home packages to online platforms. Remote learning is also possible through a variety of different channels, such as mobile phones, television, radio, and tutors. (Muñoz-Najar et al., 2021, p. 16).

Since most forms of remote learning require access to and the use of several learning or communication technologies that have not been widely adopted in the educational context, it is likely that inequalities in remote learning quality existed. This paper seeks to understand how student perspectives on remote learning varied across and within countries.

This study builds off of a conceptual framework constructed to understand remote learning during the COVID-19 pandemic. In the framework, the World Bank (Muñoz-Najar et al., 2021) identifies three essential components for remote learning take-up and effectiveness. The first component is access to technology appropriate for the remote learning needs. Learning technology not only has to be made available to students to access remote learning, but must be appropriate for the context in which it is deployed. For instance, in areas with low internet coverage, alternative strategies to online methods for providing students access to learning are necessary (e.g., paper-based materials, television or radio broadcast lessons). The second component is teacher knowledge and skills for remote teaching. This not only requires teachers to be equipped with in-depth content knowledge and strong pedagogical skills, but also to have the ability to use and integrate technology into their teaching practices. The final component is student engagement. Keeping students engaged in learning requires interesting and engaging content as well as frequent interactions with teachers (e.g., through feedback on work). According to the framework, all three components, *Available and Suitable Technology*, *Effective Teachers*, and *Engaged Students* are necessary in order to provide a quality remote learning experience for students (Muñoz-Najar et al., 2021).

In this paper, we define remote learning quality as the successful take-up and effectiveness of remote learning. Due to variation in existing technology infrastructures, digital competencies of students and teachers, and home resources for learning, we hypothesize that there will be inequalities in remote learning quality, both across- and within-countries. However, we also hypothesize that certain school-level supports might have been implemented to mitigate these inequalities. Specifically, we seek to answer the following research questions:

1. What inequalities exist in perceived remote learning quality across- and within-countries?
2. How did countries differ in their use of school-level supports/resources aimed at improving remote learning?

To answer our research questions, we use data from the Responses to Educational Disruptions Survey (REDS) study (Meinck et al., 2022). REDS gathers perspectives from students, teachers, and principals in secondary education (grade 8) on both the impacts of and responses to the educational disruption caused by the COVID-19 pandemic. REDS collected data from 11 countries spanning Africa, Asia, the Arab Region, Europe, and Latin America. Using data from the student questionnaire, we construct measures of the essential components of remote learning to capture student perspectives on the quality of their remote learning experience. With these measures, we examine the extent to which inequalities exist within and across countries in perceived remote learning quality. We also examine responses from the school questionnaire to identify supports and resources that were provided by schools to improve the quality of their students’ remote learning. Our study focuses on seven countries: Denmark, Ethiopia, Kenya, the Russian Federation, Slovenia, the United Arab Emirates, and Uzbekistan.

## Literature review

Here, we review the literature and elaborate on and more clearly define each of the essential components of remote learning. We also explore the extent to which the literature has identified existing inequalities in these measures. We end by noting some of the supports or resources that schools or national ministries provided to educators to support each of these components and help them deliver high quality remote learning to all students.

### Available and suitable technology

The first essential component of remote learning covers two aspects of technology: availability and suitability. Before determining whether the technology is suitable, it is necessary to assess the access to technology in the first place. Access to technology is frequently seen through the lens of the so-called digital divide, which refers to the gap between those who do and those who do not have access to information and communication technologies (ICTs) (Scheerder et al., 2017; van Dijk, 2006). This includes access to reliable and affordable physical devices (e.g., computers, laptops) and Internet connectivity, which are basic requirements for remote learning that takes place online (ITU, 2021a).

According to the International Telecommunication Union (ITU, 2019), prior to the COVID-19 outbreak, 93% of the world population could, in theory, access the Internet, as they were within range of a mobile broadband or Internet service. However, there are large inequalities in access both between and within countries. For example, only 23% and 11% of households in Africa and the Commonwealth of Independent States, respectively, have access to a mobile broadband network (ITU, 2020b). Within countries, gaps exist between urban and rural areas, as Internet access at home is twice as high in urban areas (72% compared to 37%). A similar situation is seen for computer access, for which 63% of households in urban areas have access, compared to only 25% in rural areas, with developing countries’ rural areas being the most disadvantaged. The differences in Internet and computer access between urban and rural areas also vary across countries. In Africa, only 28% and 17% of urban households have access to the Internet and to computers, respectively, and these figures drop to 6% and 2% for households in rural areas. In contrast, 88% and 82% of urban households in Europe have access to the Internet and computer access, while 78% and 66% of rural households have such access. Within countries, data also shows that those without access are typically the most vulnerable groups, such as women, children and youth from disadvantaged socioeconomic backgrounds, people with disabilities, and indigenous and marginalized groups (ITU, 2020a, ITU, UNESCO, & UNICEF, 2020).

#### Beyond access: the second-level digital divide

Although 93% of the population could access the Internet, just over 53% actually use it (ITU, 2019), indicating that having the opportunity to access computers and the Internet does not necessarily mean that they will be used. Therefore, the digital divide is also seen in terms of differences in digital skills or actual use, which is refered to as the second-level digital divide (Hargittai, 2002; ITU, 2021a van Dijk, 2005; Scheerder et al., 2017; Wei et al., 2011).

According to the ITU (2020a), skills are considered one of the main barriers to Internet use. For instance, data from a representative household survey compiled by the ITU show that around 65% of the population in developing countries are not using the Internet because they do not know what it is or they do not know how to use it (ITU, 2020a). The results of ICILS 2018 show that, on average, 18% of students did not have a functional knowledge of computers (Fraillon et al., 2020), putting into question students’ readiness for the shift to remote learning brought by the pandemic. Data from ICILS 2018 also shows inequalities in students’ computer and information literacy, particularly within countries (Fraillon et al., 2020). The review by Scheerder and van Dijk (2017) shows that the main determinants of the second-level digital divide are sociodemographic (e.g., gender, urban/rural dimension, residency) and economic (e.g., SES, education level, parental education) in nature, more so than material factors (e.g., ICT access at home, access quality). Besides actual skills, some researchers use self-reported skills (i.e., self-efficacy) as a proxy variable to study the second-level digital divide (Hargittai, 2005; Zhong, 2011).

#### The suitability of technology for remote learning

For effective remote learning, the availability or access to technology is necessary but not a sufficient condition, as it also needs to be suitable for the context in which it is used (Muñoz-Najar et al., 2021). During the COVID-19 school closures, there was a variety of remote learning strategies that educational systems could implement, such as online learning solutions, radio or TV broadcasts, or even paper-based take-home packages (Meinck et al., 2022; Muñoz-Najar et al., 2021). However, some countries found themselves in a remote learning paradox when they implemented unimodal strategies for remote learning that did not match the possibilities and needs of the majority of the students (Muñoz-Najar et al., 2021). For instance, when online learning was deployed but a majority of students lacked devices, had connectivity constraints, or did not have sufficient digital skills for this type of remote learning strategy.

### Effective teachers

It is well-known from previous research that teachers matter for student learning outcomes. Although measuring the impact of teachers is not straightforward, there seems to be some consensus about the importance of instructional quality. In fact, instructional quality has been regarded as the most important variable for student learning outcomes (Creemers & Kyriakides, 2008; Hattie, 2009; Scherer & Nilsen, 2016), more so than other teacher characteristics such as their qualifications or background. In other words, what matters more for student outcomes is what teachers actually do in the classrooms, as it is inside the classroom where teachers and students interact and where learning ultimately takes place (Creemers & Kyriakides, 2008; Goe, 2007). Therefore, we focus on instructional quality as a measure of effective teachers.

Instructional quality is regarded as a multidimensional construct (e.g., Fauth et al., 2014; Klieme et al., 2009; Nilsen et al., 2016), and one of its key aspects is supportive climate. A supportive classroom climate covers aspects of teacher-student interaction, such as providing positive and constructive feedback, having a positive approach to students’ errors and misconceptions, providing extra help when needed, listening and respecting students’ ideas and questions, and caring about and encouraging students (Blömeke et al., 2016; Klieme et al., 2009). Across the literature, there is evidence that a supportive climate affects student engagement and, consequently, student achievement (Berkowitz et al., 2017). The review by Berkowitz et al. (2017) also shows that a positive school climate—which includes classroom supportive climate—decreases the correlation between SES and student achievement. Bergem et al. (2020), based on Norwegian data, also find that instructional quality, including aspects related to teacher support, is important for students’ motivation to learn mathematics, particularly for low-SES students.

### Engaged students

Effective remote learning also requires student engagement. According to Skinner et al. (2009), in general, engagement refers to “the quality of a student’s connection or involvement with the endeavor of schooling and hence with the people, activities, goals, values, and place that compose it” (p. 494). Across the literature, student engagement includes aspects such as motivation to learn, emotional responses to the learning activities and the learning environment (e.g., towards teachers, classmates, school), absence of disruptive behavior, and involvement in learning tasks (e.g., paying attention, asking questions, contributing to class discussion) (Fredricks et al., 2004; Fredricks et al., 2012; Salas-Pilco et al., 2022).^1^

Student engagement is considered an important educational outcome by itself (Moore et al., 2015) but research also shows that it is a crucial factor in predicting academic achievement (Alrashidi et al., 2016) and influencing other outcomes, such as emotional well-being (Subramainan & Mahmoud, 2020). Engagement is recognized to be highly influenced by contextual factors, representing an attractive and potentially malleable influence for student learning (Fredricks et al., 2004). Examples of such factors are good-quality teacher-student relationships (Quin, 2017), and teacher and parent support (Lam et al., 2016; Wang & Eccles, 2012). In the context of the pandemic, the World Bank highlights access to suitable remote learning technology, access to engaging content, regular feedback and motivation from teachers, and a suitable home learning environment as factors that can influence the engagement of students (Muñoz-Najar et al., 2021). Additionally, researchers have highlighted the role of self-efficacy for motivation and engagement (Schunk & Mullen, 2013).

Regarding equity in student engagement, specific subgroups of students are at higher risk of suffering low levels of engagement (OECD, 2013). For example, previous research suggests boys tend to show lower engagement (Lam et al., 2016; Lietaert et al., 2015; OECD, 2013). However, educational barriers disproportionately affect girls in developing countries due to increased domestic responsibilities and gender bias, which could also affect their behavioral, emotional and cognitive engagement. Results from the OECD (2013) also suggest that students from low socioeconomic status are also more likely to show lower levels of engagement.

### School supports during the COVID-19 educational disruption

As the COVID-19 pandemic led to widespread closures of schools, educational systems were faced with the unprecedented challenge of continuing learning without the typical resources that are available in in-person learning environments. In response, national ministries or local governments overseeing schools provided guidelines for strategies to support learning continuity during the educational disruption (American Institutes for Research, 2021; Barron Rodriguez et al., 2021; Stancel-Piatak et al., 2022). Many of the strategies were explicitly or implicitly aimed at supporting the three essential components for remote learning success (Muñoz-Najar et al., 2021).

#### Providing access to suitable technology

Schools were advised to provide students and teachers with the tools necessary to access remote learning spaces. To reach this goal, governments would often partner with the private sector to facilitate student access to remote learning. For instance, many countries noted that they heavily subsidized the cost of internet or data plans (UNESCO, UNICEF, the World Bank, & OECD, 2021). While many schools distributed technology (e.g., laptops, broadband internet) to homes, several countries or areas within countries had limited internet coverage leaving it impossible to hold lessons in an online format. In these locations, it was necessary to make sure that other forms of instruction were made available (e.g., television and radio broadcasts, paper-based materials). For example, in Uzbekistan, teachers were encouraged to prepare video lessons that would be broadcast across six TV channels of the National TV and Radio Company. In Kenya, the Ministry of Education provided radio lessons to approximately 40 community and church radio stations to be broadcast to students. In instances where these video or radio lessons were not accessible, paper learning materials were encouraged to be distributed to homes. In all countries included in our analysis, national plans or policies for remote learning either required or recommended the physical distribution of learning materials to students (Stancel-Piatak et al., 2022).

#### School supports for effective teaching

In supporting effective teaching practices during remote learning, many schools were advised to provide professional development opportunities targeted at supporting teachers in their use of technology and delivering teaching remotely. Prior to the pandemic, several countries had begun to integrate the use of technology in teaching and learning (Fraillon et al., 2020). Therefore, some countries already had existing infrastructure in place to support the transition from in-person to remote learning. For example, in Uruguay, Plan Ceibal had been in place since 2007 aiming to increase and promote the use of digital technologies in education. During the pandemic, Plan Ceibal was adapted and strengthened its services provided to educators, including training and support in the use of virtual learning environments (Stancel-Piatak et al., 2022). A large majority of teachers in Uruguay were satisfied with the training provided by the program (Muñoz-Najar et al., 2021).

In addition to professional learning opportunities, several national guidelines explicitly stated that teachers were to be provided more opportunities to meet and collaborate with their peers (Stancel-Piatak et al., 2022). Collaboration among teachers in a school can facilitate learning and support innovation in teaching methods (Vangrieken et al., 2015). In the United Arab Emirates, educational institutions saw the potential benefits of facilitating collaboration among not just teachers, but schools. In their efforts to support remote teaching and learning during school closures, they established a peer pairing program for struggling schools to work with others who were seeing successes. Furthermore, an online platform was used for schools and teachers to share remote teaching and learning resources.

#### Supporting student engagement

Synchronous approaches to remote learning, those that allow for real-time interactions between students and teachers during lessons, provide a mode of instruction most similar to in-person classroom instruction that can promote student engagement (Muñoz-Najar et al., 2021). These types of approaches are best used in online formats. However, in situations where synchronous delivery methods are not available (i.e., those without access to online resources), schools had to find alternative strategies to keep students engaged. Schools could use multiple communication channels to engage students and families in the learning process. For example, in Peru, it was hypothesized that communication and feedback were key to ensure student engagement in learning (Muñoz-Najar et al., 2021). Teachers were encouraged to keep in touch with their students and students’ families using phone calls, text messages and social media (Accinelli, 2020). Frequent interaction between students and teachers resulted in moderate to high satisfaction rates for remote learning using asynchronous learning approaches (e.g., pre-recorded online lessons, television and radio broadcasts) (Muñoz-Najar et al., 2021).

As most students participated in their lessons from home, schools recognized the important partner parents/guardians could be in supporting student engagement in remote learning. Several organizations such as the Sesame Workshop and Lifelong Health made resources available to parents/guardians to support their child in learning from home Muñoz-Najar et al. (2021). Additionally, the majority (>50%) of teachers or teacher respondents in Denmark, Ethiopia, the Russian Federation, Slovenia, the United Arab Emirates, and Uzbekistan reported that the time they spent communicating with parents increased during the period of school closures (Chen et al., 2022).

## Methods

**Table 1:** Sample size by country

Country	N original sample	N filtered sample	N analytical sample
Denmark	1534	1444 (94%)	1231 (80%)
Ethiopia	3630	1952 (54%)	1558 (43%)
Kenya	1603	1305 (81%)	1171 (73%)
Russian Federation	3516	3357 (95%)	3207 (91%)
Slovenia	2552	2552 (100%)	2449 (96%)
United Arab Emirates	2988	2697 (90%)	2479 (83%)
Uzbekistan	2911	2570 (88%)	2519 (87%)

Filtered sample removes students who reported that they did not participate in remote learning during the disruption period. Analytical sample refers to the sample, after filtering, of students with complete cases of the Remote Learning Quality scales. Percentages within parentheses are relative to original sample and are unweighted

### Data

For this study, we use data from the Responses to Educational Disruption Survey (REDS) (Meinck et al., 2022). REDS utilized a two-stage stratified cluster sampling design^2^ to gather national samples of students, teachers, and schools. The student target population was defined as “all students enrolled in the grade that represents eight years of schooling counting from the first year of ISCED level 1” (Meyer et al., 2022, p. 22). Teacher and school target populations were those that taught or served students from the student target population.^3^ Questionnaires from REDS ask students, teachers, and principals to reflect on their experiences before, during, and after the “COVID-19 disruption”^4^, defined as the time period in which “[most] schools in a [country] closed for [the majority of students] in the last school year” (Fraillon & Stancel-Piatak, 2022, p. 11). Data for REDS was collected during the end of 2020 or the first half of 2021, therefore the “last school year” referred to the school year that included the start of 2020, when most schools in REDS participating countries decided to close down (Meinck et al., 2022).

With the exception of Burkina Faso, our sample includes all countries that administered the student questionnaire in REDS: Denmark, Ethiopia, Kenya, the Russian Federation, Slovenia, United Arab Emirates, and Uzbekistan. We removed students from our sample that responded that they did not do any schoolwork during the COVID-19 disruption; if the response was missing, we imputed the value using the modal response of students in that school, except in the case of Slovenia where the question was not administered. We dropped Burkina Faso from the analysis, since the number of students in the final sample was particularly low (16%, N=389 of the original 2474), meaning that very few students reported participating in remote learning. In REDS, several issues led to some countries not reaching the data quality standards. Denmark had a particularly low response rate (38%), while Ethiopia and Kenya were not able to collect fully random samples within schools and lacked information on the target population. Therefore, analyses with Denmark, Ethiopia, and Kenya are not considered representative of their populations and do not use sampling weights. The remaining countries—the Russian Federation, Slovenia, the United Arab Emirates, and Uzbekistan—present usual levels of data quality, with minor caveats (see Meinck et al., 2022). In addition, for our analyses we only worked with complete cases of Remote Learning Quality (RLQ) scales (see below), so analyses are comparable between scales. The original sample N, the filtered sample N and the analytical sample N for each country can be found in Table 1.

### Measures of remote learning quality

#### Factor analysis scales

All the measures of remote learning quality indicators were built by us, based on the original responses available in the REDS dataset. For this, we identified several items from REDS that map onto essential components for remote learning quality (see Table 8). We developed a measure of students’ perspectives on remote learning quality (RLQ), so we only took items from the student questionnaire. We only report measures included in the final analyses. Our exploratory factor analyses found suitability for several constructs of remote learning quality that fit within the World Bank conceptual framework. Among these items, we used the following scales:

- *Effective teachers:* Scale of perception of support provided by teachers during disruption.
- *Self-efficacy in technology use:* Scale of self-reported skills on using learning-related technology, as a measure of access to skills to use technology effectively.
- *Engaged students:* Scale of students’ experiences completing schoolwork remotely.

Within each scale, we applied a single imputation on the original items using the MICE package in R (van Buuren & Groothuis-Oudshoorn, 2011). We imputed those cases only where the student had a valid response in at least half of the items within a scale. If the student presented valid response in less than half of the items, the scale was marked as missing. For the predictive model we used the respective scale items and sociodemographic variables, specifically: gender, socioeconomic status, urbanicity, and language at home. The original N of complete cases, the number of cases imputed and the N of complete cases after-imputation can be found in Table 9.

For each of the three scales, we estimated scores based on a one-factor confirmatory factor analysis (CFA). We re-scaled the CFA scores to an international mean of 0 with a standard deviation of 1 using senate weights (i.e., giving the same weight to each country for the mean estimation). Using multi-group CFA, we tested the measurement invariance of these scales across countries. All CFA and measurement invariance estimations were done using the lavaan package in R (Rosseel, 2012), while exploratory analysis was done using Stata 16. The reliability (Cronbach’s Alpha) and measurement invariance models fit are available in Table 10. *Effective Teachers* and *Engaged Students* present scalar invariance between countries, meaning that the scales are comparable between countries. *Self-efficacy in Technology Use* present only metric invariance between countries, meaning that we cannot compare differences on latent means between countries. Self-reported measures of skills are prone to bias, as some students may undervalue their skills and others may overvalue them (Litt, 2013); it may be the case that students in some countries systematically underrate or overrate their skills, explaining the lack of scalar invariance.

#### Dichotomous indicator

In addition, on the dimension of *Available and Suitable Technology*, we constructed a dichotomous indicator of *Access to Quality Technology*; students with a positive response on this indicator means that they declared to have access to a device and that it worked well most of the time. Table 2 presents summary statistics of these Remote Learning Quality scales.

**Table 2:** Average remote learning quality scales and indicator by country

RLQ scale	DNK^a^	ETH^a^	KEN^a^	RUS	SVN	ARE	UZB
Self-efficacy in technology use	0.86 (0.02)	− 1.07 (0.04)	− 1.01 (0.06)	0.49 (0.03)	0.58 (0.03)	0.33 (0.04)	− 0.18 (0.03)
Access to quality technology	0.93 (0.01)	0.10 (0.02)	0.12 (0.02)	0.83 (0.01)	0.86 (0.01)	0.88 (0.01)	0.77 (0.03)
Effective teachers	− 0.11 (0.03)	− 0.29 (0.07)	− 0.78 (0.09)	0.03 (0.04)	0.05 (0.03)	0.43 (0.03)	0.65 (0.04)
Engaged students	− 0.09 (0.03)	− 0.73 (0.07)	-0.83 (0.09)	0.50 (0.03)	0.32 (0.03)	0.37 (0.03)	0.41 (0.04)

Standard errors are presented in parentheses. Probability weights have been applied and jackknife standard errors are presented for all countries except in Denmark, Ethiopia, and Kenya. Standard errors reported for Denmark, Ethiopia, and Kenya account for clustering of observations within schools. Due to lack of scalar invariance, averages on the *Self-Efficacy in Technology Use* scale should not be compared across countries.

*DNK* = Denmark,* ETH* = Ethiopia,* KEN* = Kenya,* RUS* = Russian Federation,* SVN* = Slovenia,* ARE* = United Arab Emirates,* UZB* = Uzbekistan

^a^Data may not be representative of target population.

### Measures of school-provided supports/resources for remote learning

The REDS school questionnaire includes several items that ask principals about the types of supports or resources that they provided to students, parents/guardians, and teachers during the disruption period. We identify items regarding the supports or resources that were tied specifically to the key components of successful remote learning: *Access to Quality Technology*, *Effective Teachers*, and *Student Engagement*. In our review, we did not locate any items that we believed to be related with improving *Self-efficacy in Technology Use*.

Each of the items was coded into a binary indicator specifying whether the school did (“Yes”) or did not (“No”) provide the support or resource. Details on how each of the items was coded can be found in Table 11. The support we found related with *Access to Quality Technology* was whether the school distributed suitable technology for remote learning access. That is, whether the school provided internet or devices to students during the disruption period. In the case of Ethiopia and Kenya, where access to technology was generally low (10% and 12%, respectively), we checked whether the school made paper materials available to students or supported students in accessing broadcast lessons over the television or radio.

We identify several items that ask whether schools increased resources to support teachers in implementing remote teaching. These resources include online teaching tools (e.g., online platforms, remote teaching tools), professional learning (e.g., professional development, resources for effective remote teaching pedagogy), and peer collaboration opportunities. Each item was coded so that it focuses only on schools that responded that they “Increased” resources. Therefore, schools that had already been investing in these types of resources prior to the disruption would not be included among the schools that we indicate as providing the support. Finally, we select several items that ask schools whether they provided supports for *Student Engagement*. The first set of items describe supports that were provided to encourage live interactions between students and teachers (e.g., offering live virtual lessons or support sessions). We also examine whether schools expected their teachers to provide students feedback on their work during the disruption period. Finally, we check whether schools provided any advice and/or materials to parents/guardians to support their child’s home learning environment.

In discussing school-provided supports or resources, we believe it is important to note that schools may have been constrained in their ability to provide certain resources for their students, teachers, and communities based on the priorities of higher level governments in their educational systems. For example, some schools may have only been able to provide technology for learning to their students as a result of Ministries of Education or local governments providing schools with the funds or resources to provide these services. Therefore, we note the importance of other actors in schools’ ability to provide these supports/resources for remote learning.

### Sociodemographic variables

We also use several student and school background variables in our analysis. First, we use a measure of students’ socioeconomic status (SES). The scale, available in the REDS dataset, was constructed using information about the number of learning resources (e.g., a quiet place to work, ICT devices) and books in the home, parents’ level of education and occupation, and language spoken at home (UNESCO & IEA, 2022). The scale is divided into three categories based on tertiles on the continuous scale across countries: high SES (top tertile), medium SES (middle tertile), and low SES (bottom tertile). Second, we use information about where the school is located. Specifically, we distinguish between schools in a “Big town or city” (located in a town with $\ge$ 15,000 people) and those in a small town (located in town with < 15,000 people). This was a similar way to how school locations were categorized in Strietholt and Süttmann (2022). Throughout the report we also use “urban” and “rural” to distinguish between schools located in a big town or city and a small town, respectively. Third, we look at student-reported gender (i.e., Boy, Girl).^5^ Finally, we look at student home language and whether it differs from the language of instruction. Specifically, students were asked “What language do you speak at home most of the time?” In relation to this item’s responses, it is important to note that, in Kenya, the language of instruction is English for most students, yet this often does not match with the language most often spoken in the catchment area (Dexis Consulting Group, 2021). Hence, there is a large proportion of students speaking a language other than the language of instruction. This is important for interpretation of results by language in Kenya. Table 3 shows a summary of these variables for our analytic sample by country.

**Table 3:** Overview statistics of students by country

	DNK^a^	ETH^a^	KEN^a^	RUS	SVN	ARE	UZB
Socioeconomic status							
Low	4% (0.6%)	78% (1.0%)	77% (1.2%)	5% (1.1%)	5% (0.6%)	3% (0.6%)	14% (1.7%)
Medium	44% (1.4%)	19% (1.0%)	18% (1.1%)	52% (1.7%)	46% (1.6%)	41% (2.1%)	63% (1.7%)
High	48% (1.4%)	3% (0.4%)	4% (0.5%)	42% (2.2%)	49% (1.8%)	55% (2.2%)	24% (1.7%)
*Missing*	4% (0.6%)	0% (0.1%)	1% (0.3%)	0% (0.1%)	0% (0.1%)	1% (0.3%)	0% (0.1%)
Urbanicity							
Small town	42% (1.4%)	69% (1.2%)	79% (1.2%)	30% (4.1%)	72% (5.5%)	22% (3.7%)	82% (4.1%)
Big town or city	20% (1.1%)	20% (1.0%)	13% (1.0%)	69% (3.7%)	22% (4.9%)	73% (4.0%)	18% (4.1%)
*Missing*	38% (1.4%)	11% (0.8%)	8% (0.8%)	1% (1.1%)	6% (2.8%)	6% (2.2%)	0% (0.0%)
Gender							
Boy	40% (1.4%)	53% (1.3%)	47% (1.5%)	49% (1.1%)	50% (1.3%)	46% (3.5%)	49% (1.4%)
Girl	51% (1.4%)	43% (1.3%)	49% (1.5%)	51% (1.1%)	49% (1.3%)	54% (3.5%)	51% (1.4%)
Other	4% (0.5%)	–	0% (0.1%)	–	–	–	–
*Missing*	5% (0.6%)	4% (0.5%)	4% (0.5%)	0% (0.0%)	1% (0.9%)	0% (0.0%)	0% (0.0%)
Home language							
Lang. of instruction	88% (0.9%)	46% (1.3%)	3% (0.5%)	91% (2.1%)	88% (1.4%)	72% (2.4%)	93% (2.2%)
Other language	7% (0.7%)	50% (1.3%)	93% (0.7%)	9% (2.1%)	11% (1.4%)	27% (2.5%)	7% (2.2%)
*Missing*	5% (0.6%)	3% (0.5%)	4% (0.5%)	0% (0.1%)	1% (0.2%)	2% (0.5%)	0% (0.1%)
Total N	1231	1558	1171	3207	2449	2479	2519

Standard errors are presented in parentheses. Probability weights have been applied and jackknife standard errors have been calculated in all countries except in Denmark, Ethiopia, and Kenya.

*DNK* = Denmark, *ETH* = Ethiopia, *KEN* = Kenya, *RUS* = Russian Federation, *SVN* = Slovenia, *ARE* = United Arab Emirates, *UZB* = Uzbekistan

^a^Data may not be representative of target population.

### Analytic approach

We follow the suggested procedures for analyzing data from the REDS international database (UNESCO & IEA, 2022). That is, we apply probability weights and estimate variance using the jackknife repeated replication (JRR) method, where possible. JRR incorporates the sampling variance within the standard error estimations; this step is needed due the complex sampling and the clustering of students within schools (González & Foy, 2009). Due to some countries not reaching data quality standards, the use of weights and JRR was not possible in three of our seven selected countries (Denmark, Ethiopia, and Kenya). In regression models, standard errors for these countries have been calculated accounting for clustering of observations at the school level (Rogers, 1994). However, weights have not been applied so results should not be interpreted as representative of the target population.

To explore inequalities in perceived remote learning quality within countries, we test differences in our RLQ scales by several student characteristics: socioeconomic status, urbanicity, gender, and home language. We conduct these tests in a regression framework. Specifically, we estimate, separately for each country, regression models of the following form:

1 $$\begin{aligned} RLQ_{i} = \alpha + \beta Grp_{i} + \varepsilon _i \end{aligned},$$

where $RLQ_{i}$ is one of our measures of perceived remote learning quality for student *i* and $Grp_{i}$ is some categorical variable representing a characteristics of student *i* (i.e., girl vs. boy, high SES vs. low SES, speaks language of instruction vs. other language at home) or student *i*’s school (located in an big town or city vs. small town). The coefficient $\beta$ tests the difference in average perceived remote learning quality across student groups within a country. A significant coefficient would be indicative of inequalities in perceived remote learning quality within a country.

All analyses were done using Stata 16, taking advantage of its support of complex samples on survey analyses (i.e., jackknife).

## Results

### Across-country inequalities in student perceptions of remote learning quality

**Fig. 1:**
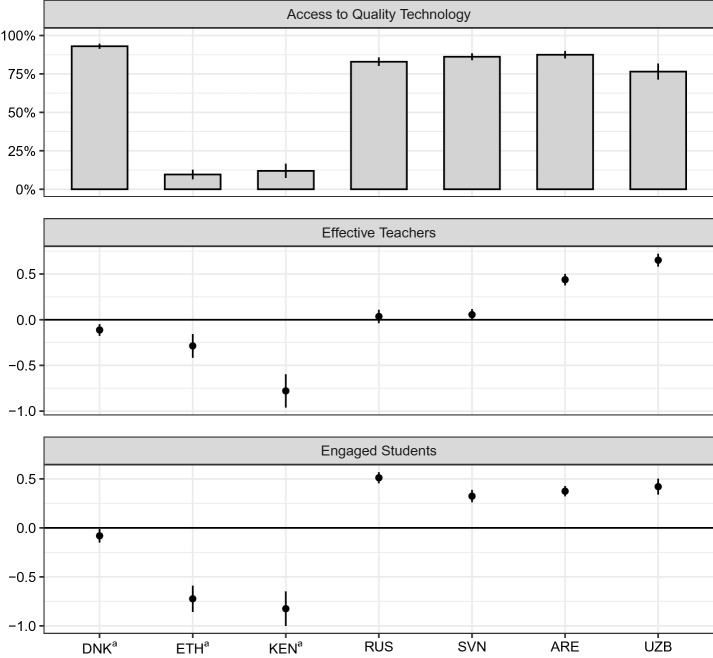
Cross-country Comparisons of Remote Learning Quality Scales and Indicator. ^a^Data may not be representative of target population. Notes: 95% confidence intervals are presented as error bars around mean estimates. The *Access to Quality Technology* indicator (top panel) shows the share of students with a positive value on the measure. Each scale (second and third panel) has an international average of 0 (represented by the horizontal line) and a standard deviation of 1 after applying senate weights. Probability weights have been applied and jackknife standard errors have been calculated in all countries except in Denmark, Ethiopia, and Kenya. Standard errors calculated for Denmark, Ethiopia, and Kenya account for clustering of observations within schools. Due to lack of scalar invariance, averages on the *Self-Efficacy in Technology Use* scale are not presented. *DNK* = Denmark, *ETH* = Ethiopia, *KEN* = Kenya, *RUS* = Russian Federation, *SVN* = Slovenia, *ARE* = United Arab Emirates, *UZB* = Uzbekistan

We begin our exploration by examining cross-country differences in our remote learning quality (RLQ) measures. In interpreting the results, it is important to note that data from Denmark, Ethiopia, and Kenya may not be representative of the target population and only reflect findings for student respondents on the REDS questionnaire. Furthermore, given a lack of scalar invariance in our *Self-Efficacy in Technology Use* scale, we do not present means for this scale in Fig. 1 as they cannot be compared across countries.

A key component for successful remote learning is giving students access to the devices or content needed to continue learning outside of a school setting. The *Access to Quality Technology* indicator captures the share of students who report that they had both a good quality device (e.g., a laptop or tablet) as well as reliable internet access. The top panel of Fig. 1 shows the summary of the *Access to Quality Technology* indicator across countries. Unlike the scales, which are continuous, the mean values should be interpreted as the share of students responding that they had access to quality technology for remote learning. While most countries had at least three quarters of students or student respondents saying that they had access to quality devices, both Ethiopia and Kenya stood out, with only 10% and 12% of student respondents having access to quality devices or internet, respectively. It should be noted that, in results not shown here, despite these low levels of access, many students respondents in Ethiopia and Kenya reported that schools provided other means for them to access content, either through paper-based worksheets or lessons broadcast on the television or radio (84% and 92%, respectively). So while it appears that Ethiopia and Kenya did not have the technology to provide remote learning online, other methods were used to reach their students.

In addition to having access to technology, students also need effective teachers that can support them in their learning in a remote setting. The second panel of Fig. 1 compares means across countries on the *Effective Teachers* scale. The horizontal line in this panel represents the international average for the included countries. Uzbekistan and the United Arab Emirates had the highest values on the scale (0.65 and 0.43, respectively), indicating that students in these countries felt more supported by their teacher during the disruption than students in other countries. Ethiopia and Kenya stand out with particularly low values on the *Effective Teachers* scale ($-0.29$ and $-0.78$, respectively).

As well as access to suitable technology and effective teachers, students need to be engaged during remote learning in order for it to be successful. The bottom right panel of Fig. 1 compares means on the *Engaged Students* scale across countries. The Russian Federation, Slovenia, the United Arab Emirates, and Uzbekistan all had averages significantly above the international average on this measure of student engagement. Ethiopia and Kenya had the lowest average values on the scale, − 0.73 and − 0.83, respectively.

### Within-country inequalities in student perceptions of remote learning quality

**Table 4:** Inequalities in self-efficacy in technology use scale

	DNK^a^	ETH^a^	KEN^a^	RUS	SVN	ARE	UZB
Socioeconomic Status: Baseline = “Low”							
Medium	− 0.01 (0.10)	0.53* (0.06)	0.40* (0.06)	0.34* (0.09)	0.17 (0.12)	0.30* (0.10)	0.21* (0.07)
High	0.08 (0.11)	0.98* (0.17)	1.47* (0.27)	0.51* (0.10)	0.21 (0.12)	0.60* (0.11)	0.42* (0.09)
N	1179	1554	1161	3205	2446	2460	2518
Urbanicity: Baseline = “Small town”							
Big town or City	0.01 (0.06)	0.32* (0.12)	0.57* (0.23)	0.20* (0.05)	− 0.04 (0.07)	0.23* (0.09)	0.14 (0.08)
N	760	1393	1083	3181	2326	2335	2519
Gender: Baseline = “Boy”							
Girl	− 0.37* (0.04)	− 0.12* (0.04)	− 0.16* (0.08)	-0.22* (0.04)	− 0.36* (0.04)	-0.16* (0.06)	− 0.14* (0.04)
N	1173	1497	1129	3207	2439	2479	2519
Home Language: Baseline = “Language of Instruction”							
Other language	0.03 (0.07)	− 0.11 (0.07)	-0.26* (0.12)	− 0.24* (0.08)	− 0.11 (0.07)	0.21* (0.06)	0.01 (0.10)
N	1171	1505	1128	3197	2430	2431	2513

Coefficients for groups come from separate regressions. Standard errors shown in parentheses. Probability weights have been applied and jackknife standard errors have been calculated in all countries except in Denmark, Ethiopia, and Kenya. In Denmark, Ethiopia, and Kenya standard errors have been clustered by school

*DNK* = Denmark, *ETH* = Ethiopia, *KEN* = Kenya, *RUS* = Russian Federation, *SVN* = Slovenia, *ARE* = United Arab Emirates, *UZB* = Uzbekistan

^a^Data may not be representative of target population

* p < 0.05

In this part of the results, we explore within-country inequalities in the remote learning quality measures across several different sociodemographic groups. Table 4 displays estimated differences in the *Self-efficacy in Technology Use* scale across several groupings, within each country. Inequalities between medium or high SES and low SES students in self-efficacy in technology use is evident across six of the seven countries. The scale is standardized so the size of the differences can be interpreted in standard deviations. The inequalities range in size from 0.21 to 1.47 standard deviations on the scale. The largest gaps appear for responding students in Ethiopia (0.98 difference between high and low SES, 0.53 difference between medium and low SES) and Kenya (1.47 difference between high and low SES, 0.40 difference between medium and low SES). The gaps indicate that student confidence in their ability to use technology is significantly higher for students in families with more resources. In contrast, in Denmark and Slovenia, we find no significant differences between medium or high SES students and low SES students. That would indicate that student respondents across Denmark and Slovenia tended to be confident in using technology for learning, regardless of their home resources.

In comparing the *Self-efficacy in Technology Use* scale between students attending school in a big town or city versus a school in a small town, we observe significant differences in favor of students in more urban schools for four of the seven countries. The size of the inequalities ranged between 0.20 to 0.57 standard deviations. The largest differences appear in Ethiopia and Kenya. While most student respondents were in schools that reported being located in a small town, it appears that the few students living in more urban areas had greater confidence in their abilities to use technology entering the pandemic (see Table 3). In contrast, Denmark, Slovenia, and Uzbekistan did not show results that indicated inequalities in the *Self-efficacy in Technology Use* scale between urban and rural schools.

Girls tended to report lower levels of confidence in their ability to use technology than boys across all countries. The size of the inequality ranged between 0.12 and 0.37 standard deviations. The largest differences are found in Denmark and Slovenia, where boys, on average, score about 0.37 and 0.36 points higher than girls, respectively, on the *Self-efficacy in Technology Use* scale.

In exploring differences by students’ home language, we find significant gaps in three of the seven countries. The largest differences show that students who speak a different language than the language of instruction at home tended to have lower levels of confidence in using technology. These gaps were largest and significant in Kenya and the Russian Federation ($-0.26$ and $-0.24$, respectively). However it is important to recall that the majority of student respondents in Kenya (93%) reported that they spoke a language other than the language of instruction (English) at home showing that the few students who did speak English at home had higher confidence than those who did not. The opposite pattern was found in the United Arab Emirates, where students speaking the language of instruction scored 0.21 points lower on the *Self-efficacy in Technology Use* scale than their counterparts. The difference was not significant in the other countries.

**Table 5:** Inequalities in *access to quality technology* indicator

	DNK^a^	ETH^a^	KEN^a^	RUS	SVN	ARE	UZB
Socioeconomic status: baseline = “Low”							
Medium	0.00 (0.04)	0.15* (0.04)	0.20* (0.04)	0.18* (0.05)	0.04 (0.06)	0.07 (0.06)	0.26* (0.06)
High	− 0.01 (0.04)	0.38* (0.10)	0.63* (0.14)	0.24* (0.05)	0.09 (0.06)	0.13* (0.06)	0.37* (0.06)
N	1179	1554	1161	3205	2446	2460	2518
Urbanicity: baseline = “Small town”							
Big town or City	− 0.00 (0.02)	0.10 (0.05)	0.19 (0.10)	0.06 (0.03)	− 0.01 (0.03)	0.08* (0.03)	0.10* (0.05)
N	760	1393	1083	3181	2326	2335	2519
Gender: baseline = “Boy”							
Girl	− 0.02 (0.01)	0.01 (0.02)	− 0.03 (0.04)	0.02 (0.02)	0.04* (0.02)	− 0.02 (0.02)	− 0.02 (0.03)
N	1173	1497	1129	3207	2439	2479	2519
Home language: baseline = “Language of Instruction”							
Other language	− 0.05 (0.03)	− 0.03 (0.03)	− 0.17* (0.08)	− 0.08 (0.05)	− 0.05 (0.03)	0.01 (0.02)	− 0.09 (0.08)
N	1171	1505	1128	3197	2430	2431	2513

Coefficients for groups come from separate regressions. Standard errors shown in parentheses. Probability weights have been applied and jackknife standard errors have been calculated in all countries except in Denmark, Ethiopia, and Kenya. In Denmark, Ethiopia, and Kenya standard errors have been clustered by school.

*DNK* = Denmark, *ETH* = Ethiopia, *KEN* = Kenya, *RUS* = Russian Federation, *SVN* = Slovenia, *ARE* = United Arab Emirates, *UZB* = Uzbekistan

^a^ Data may not be representative of target population

*p < 0.05

We next examine inequalities in access to quality technology within each country. Table 5 shows the estimated differences in the *Access to Quality Technology* indicator across student and school groups. When comparing high or medium SES students with low SES students, inequalities were found in five of the seven countries. In the five countries, high or medium SES students were significantly more likely to respond to having a quality device and reliable internet access (an exception was in the United Arab Emirates where there was no significant difference between medium SES and low SES students). Student respondents in Kenya and Ethiopia reported having generally low levels of quality technology access compared to the other countries (10% and 12%, respectively) and the observed gap may reflect limited resources. That is, schools in these countries may not have had the ability to provide nearly 90% of their students with a good quality device or internet coverage. However, in results not shown here, we still observe inequalities in student access to other types of remote learning materials (e.g., paper-based assignments or television/radio broadcast lessons) in Ethiopia and Kenya. Specifically, high and medium SES students were significantly more likely to have access to these alternative resources for remote learning compared to low SES students (with one exception in Kenya, where high SES students did not have significantly higher access to these materials compared to low SES students). However, higher levels of technology access did not necessarily mean that all students had access as illustrated by the observed inequalities in the Russian Federation and Uzbekistan. In contrast, however, Denmark and Slovenia, also with high levels of technology access, showed no significant gaps in technology access across socioeconomic levels.

Schools located in a big town or city had higher shares of students reporting having access to quality device in the United Arab Emirates and Uzbekistan. However, it could be argued that there were large differences across school locations in Ethiopia, Kenya, and the Russian Federation (although the findings are not significant at the 5% level, only at the 10%). Findings may be a result of generally poor internet coverage in rural areas (ITU, 2020b; 2021b). If these countries have the resources to provide technology to students, the findings indicate that it would be worth investing in schools located in small towns as they were less likely to have students with good quality devices or internet coverage.

When comparing the *Access to Quality Technology* indicator by gender and home language, relatively fewer inequalities were observed. One exception was in Slovenia, where a higher share of girls had access to technology than boys did. Another exception was in Kenya where the share of students speaking a language other than the language of instruction at home (the majority) was 17 percentage points lower than their counterparts. That is, those who spoke English at home in Kenya were more likely to have access to quality technology as well as have confidence in their ability to use technology (see Table 4).

**Table 6:** Inequalities in effective teachers scale

	DNK^a^	ETH^a^	KEN^a^	RUS	SVN	ARE	UZB
Socioeconomic status: baseline = “Low”							
Medium	0.01 (0.13)	0.01 (0.10)	0.41* (0.11)	− 0.08 (0.09)	− 0.11 (0.15)	0.12 (0.15)	0.17* (0.07)
High	0.02 (0.14)	− 0.29 (0.18)	1.48* (0.66)	− 0.08 (0.11)	− 0.13 (0.15)	0.12 (0.15)	0.34* (0.08)
N	1179	1554	1161	3205	2446	2460	2518
Urbanicity: baseline = “Small town”							
Big town or city	− 0.13 (0.10)	− 0.14 (0.18)	0.78* (0.34)	− 0.06 (0.09)	− 0.09 (0.08)	− 0.08 (0.07)	− 0.02 (0.13)
N	760	1393	1083	3181	2326	2335	2519
Gender: baseline = “Boy”							
Girl	− 0.12* (0.05)	− 0.00 (0.07)	− 0.13 (0.12)	− 0.11* (0.05)	0.01 (0.06)	− 0.02 (0.07)	0.15* (0.04)
N	1173	1497	1129	3207	2439	2479	2519
Home language: baseline = “Language of Instruction”							
Other language	− 0.19 (0.11)	0.37* (0.12)	− 0.08 (0.18)	0.10 (0.11)	0.03 (0.09)	− 0.21* (0.06)	0.02 (0.11)
N	1171	1505	1128	3197	2430	2431	2513

Coefficients for groups come from separate regressions. Standard errors shown in parentheses. Probability weights have been applied and jackknife standard errors have been calculated in all countries except in Denmark, Ethiopia, and Kenya. In Denmark, Ethiopia, and Kenya standard errors have been clustered by school

*DNK* = Denmark, *ETH* = Ethiopia, *KEN* = Kenya, *RUS* = Russian Federation, *SVN* = Slovenia, *ARE* = United Arab Emirates, *UZB* = Uzbekistan

^a^Data may not be representative of target population

*p < 0.05

Inequalities in student perceptions of their teachers’ effectiveness during the disruption period are examined in Table 6. Across the socioeconomic status, school urbanicity, gender, and home language groupings, most countries do not show significant inequalities in the perceived effectiveness of teachers. That is, students had the same level of perceived support from their teachers across most countries, regardless of their socioeconomic background, gender, language status, or school location. Exceptions to this pattern were Kenya and Uzbekistan when comparing differences across different SES levels. In these countries, high or medium SES students had higher values on the *Effective Teachers* scale compared to low SES students. Kenya was the exception when examining differences by school location, having higher perceptions of teacher effectiveness in schools located in a big town or city. Denmark, the Russian Federation, and Uzbekistan all showed inequalities by gender, with boys having higher scores on the *Effective Teachers* scale than girls in Denmark and the Russian Federation and the opposite being true in Uzbekistan. Finally, Ethiopia and the United Arab Emirates had significant inequalities across home language groups. In the United Arab Emirates, students speaking the language of instruction had higher perceptions of their teacher’s effectiveness than their counterparts. The opposite pattern was observed in Ethiopia. Despite the observed inequalities in the *Self-Efficacy in Technology Use* scale, it appears the perceptions of effective teaching were mostly the same within countries with the noted exceptions.

**Table 7:** Inequalities in student engagement scale

	DNK^a^	ETH^a^	KEN^a^	RUS	SVN	ARE	UZB
Socioeconomic status: baseline = “Low”							
Medium	− 0.21 (0.14)	0.64* (0.08)	0.56* (0.10)	0.25* (0.12)	− 0.03 (0.12)	0.10 (0.12)	0.17* (0.08)
High	− 0.21 (0.15)	0.91* (0.15)	1.12* (0.19)	0.30* (0.12)	− 0.03 (0.12)	0.01 (0.12)	0.33* (0.10)
N	1179	1554	1161	3205	2446	2460	2518
Urbanicity: baseline = “Small town”							
Big town or City	0.04 (0.10)	0.15 (0.15)	0.72* (0.18)	0.02 (0.05)	− 0.04 (0.09)	− 0.03 (0.07)	0.03 (0.08)
N	760	1393	1083	3181	2326	2335	2519
Gender: baseline = “Boy”							
Girl	− 0.25* (0.06)	0.13* (0.06)	− 0.02 (0.08)	− 0.02 (0.04)	− 0.16* (0.05)	− 0.09 (0.06)	0.09 (0.05)
N	1173	1497	1129	3207	2439	2479	2519
Home language: baseline = “Language of Instruction”							
Other language	0.07 (0.11)	− 0.28* (0.13)	− 0.35* (0.17)	− 0.12 (0.12)	− 0.03 (0.10)	− 0.10 (0.07)	0.11 (0.09)
N	1171	1505	1128	3197	2430	2431	2513

Coefficients for groups come from separate regressions. Standard errors shown in parentheses. Probability weights have been applied and jackknife standard errors have been calculated in all countries except in Denmark, Ethiopia, and Kenya. In Denmark, Ethiopia, and Kenya standard errors have been clustered by school

*DNK* = Denmark, *ETH* = Ethiopia, *KEN* = Kenya, *RUS* = Russian Federation, *SVN* = Slovenia, *ARE* = United Arab Emirates, *UZB* = Uzbekistan

^a^Data may not be representative of target population

*p < 0.05

We finally explore within-country inequalities in student engagement. Table 7 presents tests for inequalities in the *Student Engagement* scale. In comparing high or medium SES with low SES students, we find significant differences in four out of seven countries. In those four countries, high or medium SES students tended to have higher values on the *Student Engagement* scale compared to low SES students. Inequalities ranged from 0.17 to 1.12 standard deviations. The largest differences were observed in Ethiopia and Kenya, where high SES student respondents had averages on the *Student Engagement* scale 0.91 and 1.12 standard deviations higher than low SES students, respectively (or 0.64 and 0.56 standard deviation differences when comparing medium SES to low SES students, respectively). Further differences in student engagement were found when comparing by gender. Three out of seven countries had significant differences between boys and girls. In Denmark and Slovenia, boys had higher values on the *Student Engagement* scales than girls. The opposite was true in Ethiopia, where girls had higher engagement measures than boys.

Relatively fewer differences were found when comparing across home language and school location. When comparing across home language groups, student respondents in Ethiopia and Kenya had significantly lower reported levels of student engagement among students speaking a language other than the language of instruction. Finally, schools located in a big town or city had students with higher levels of engagement than those in schools located in a small town. However, these were exceptions. Most differences were not found to be significant when comparing across these student groupings.

### School-provided supports/resources for remote learning

**Fig. 2:**
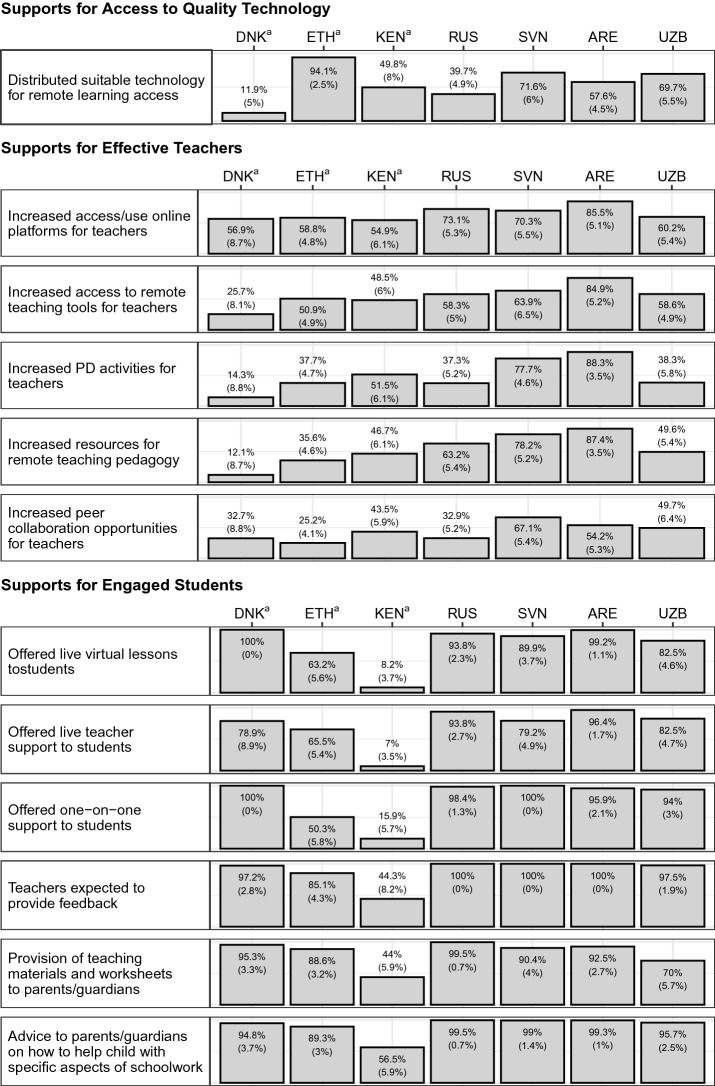
Cross-country Comparisons of School Supports and Resources for Remote Learning. ^a^Data may not be representative of target population. Notes: Standard errors are presented in parentheses. Probability weights have been applied and jackknife standard errors are presented for all countries except in Denmark, Ethiopia, and Kenya. Standard errors reported for Denmark, Ethiopia, and Kenya account for clustering of observations within schools. *DNK* = Denmark, *ETH* = Ethiopia, *KEN* = Kenya, *RUS* = Russian Federation, *SVN* = Slovenia, *ARE* = United Arab Emirates, *UZB* = Uzbekistan

We finally examine the extent to which resources were provided by schools to students, teachers, or parents/guardians in order to support remote learning implementation. The first row of Fig. 2 shows the share of students in schools that provided at least some students with access to remote learning. That is, these are schools that provided laptops and internet access to at least some of their students. In Ethiopia and Kenya, given the low levels of technology access, we look at schools who made paper-based materials or television/radio broadcast lessons available. While near to over half of students were in schools that reported providing some form of suitable technology during the disruption period in five of the seven countries, the distribution of technology is not a necessary component to ensure access. For instance, in a country like Denmark, over 90% of students respondents indicated that they had a quality device and reliable internet access. It would not be necessary for schools to provide devices or internet to students when most students already had access to a device. The distribution of suitable technology would be most appropriate in areas where there are already low levels of access or inequalities are present so that schools can target their resources to students most in need. Therefore, it is promising to see so many schools in Ethiopia making materials available to students so that they are able to continue to access lessons outside the classroom. Furthermore, we highlight the relatively high level of distribution that occurred in Slovenia’s schools (71.6%). In comparing with our other findings, data from Slovenia did not reveal any significant inequalities in our *Access to Quality Technology* indicator. However, the same pattern did not appear in the data for Uzbekistan, where the high use of this strategy (69.7%) was not paired with findings of no inequalities in our technology access indicator.

The next section of Fig. 2 shows whether resources were increased to support teachers during the disruption period. Given the wording of these questions, it is important to note that some schools might have provided sufficient resources for these teacher supports prior to the disruption period. Therefore, a school not increasing the use of these supports during the disruption period does not necessarily mean that it was ignored, it just highlights the schools where extra efforts were made to focus on such practices. The findings show a good range of variation in whether schools increased or did not increase such supports. Schools in Slovenia and the United Arab Emirates generally responded that they increased the access and level of specific supports or resources for teachers in their schools (above 50% across the listed teacher supports). When comparing this with the inequalities in the *Effective Teachers* scale, few inequalities were found in Slovenia and the United Arab Emirates (the only exception being students who speak another language than the language of instruction at home had significantly lower perceptions of their teachers’ effectiveness in the United Arab Emirates).

The final section of Fig. 2 shows the use of several strategies used to promote student engagement. The first set of strategies, offering live lessons or support, tended to be used in most countries where online remote learning was possible (i.e., all countries except in Ethiopia and Kenya where there were very low levels of technology access). Expecting teachers to provide feedback to students was generally found to be the case in all countries except Kenya (all above 85%). Finally, schools also provided resources to parents/guardians to help them in supporting their child with remote learning. The majority of students across all countries were in schools that provided this support to parents. One exception was in Kenya, where only 44% of student respondents were in schools that reported that they provided teaching materials and worksheets to parents and guardians and only 57% of student respondents were in schools that reported that they provided advice to parents/guardians on how to help with specific aspects of schoolwork.

#### Additional analyses on the use of school-provided supports/resources for remote learning

We performed additional analyses estimating inequalities in the use of these supports within countries. However, results did not reveal many significant or meaningful differences. In addition, regression analysis was done to test the association between a school’s use of these supports and our measures of remote learning quality. However, we did not find many significant associations. Given the lack of significant results and the fact that we believe the analysis was outside of the scope of the current study, we do not report these results.

## Discussion and conclusion

Using student perceptions of their remote learning experience, we develop several measures of remote learning quality proposed by the World Bank to assess remote learning success: *Self-efficacy in Technology Use*, *Access to Quality Technology*, *Effective Teachers*, and *Engaged Students* (Muñoz-Najar et al., 2021). Findings reveal several inequalities across countries in our sample. Specifically, in the African countries, Ethiopia and Kenya, student respondents reported much lower levels of access to quality technology needed to access online lessons. Not only that, but a considerably low proportion of student respondents in the three African countries that participated in REDS reported that they did not participate in remote learning during the disruption (see Table 1), especially in Ethiopia and Burkina Faso (which had to be dropped from the analysis because of this). This result already indicates some disparities across countries in the access to remote learning, reflecting the challenges in the implementation of remote learning in some countries. These results show that not all countries were prepared to offer online instruction to students. However, we also find that Ethiopia and Kenya found other ways to provide access to learning to their students (i.e., by providing paper-based worksheets and learning materials or broadcasting lessons over the television or radio). Despite this, Ethiopia and Kenya, still had relatively lower scores on our *Effective Teachers* and *Engaged Students* scales. While this might be concerning, it likely reveals the advantages of online synchronous instruction that allows teachers to provide more tailored and personal support to students and gives more opportunities to engage students in lessons. Unfortunately, it seems from our findings, that countries where students were extremely limited in their access to technology were unable to offer the type of remote learning experience that would engage students and showcase the effectiveness of teachers. However, we also find that having access to technology was not sufficient on its own to ensure more effective teachers or higher student engagement as illustrated in data from Denmark.

Within countries, our findings reveal that, in many of the countries examined, inequalities in student access to and confidence in using technology presented challenges in implementing remote learning for certain groups of students. In line with the literature on the digital divide, these groups were, typically, low-SES students, girls^6^, or students living in more rural areas. This was the case even in some countries that had high overall levels of access to technology (e.g., the Russian Federation, the United Arab Emirates, and Uzbekistan). These findings indicate that, even in countries that may have high levels of access to learning technologies, there were still some groups of students that could use additional support in gaining access to technology as well as help on how to use it. Targeted distribution could be one way to address these concerns of within-country inequalities. That is, schools could identify student technological needs and make devices and internet coverage available to them as well as resources and help on how to use them. For this to be most effective, baseline measures in technology access or technological skills would assist in identifying students most in need of support.

These inequalities in countries’ ability to offer online remote learning may have carried over into inequalities in student engagement. As referenced earlier, synchronous activities (usually facilitated by online interaction) were one mechanism to keep students engaged even outside the classroom (Muñoz-Najar et al., 2021). A lot of the inequalities in student engagement found within countries were associated with inequalities in access to quality technology, illustrating a possible consequence of the digital divide in educational systems forced to transition learning into a remote setting.

Despite the challenges observed within countries, student perceptions of the effectiveness of their teachers, generally, did not differ significantly across these groups (with some exceptions noted in the Results section). Taken together, these findings imply that, within-countries, schools were not hindered (nor helped) in their efforts to prepare teachers for managing classrooms remotely by limitations in technology access or use. Within countries, students with lower levels of access and confidence in their skills to use technology, perceived their teachers in similar ways to their counterparts. Whether this is because of the additional effort made by teachers in these countries to ensure the continuity of learning in a fair and equitable way or because students, who are closer to teachers, more recognized their efforts, rather than the supports provided at higher levels (i.e., school or institutional policies) cannot be answered from our analysis. We feel that future research could further explore the implications of this finding.

The relatively lower number of inequalities in our *Effective Teachers* measure may be related to the efforts that schools made to support their students’ remote learning experience. In our review of several school-provided supports or resources for remote learning, we find that increased efforts to support teachers evident in Slovenia and the United Arab Emirates was paired with fewer inequalities in these measures. In addition, many schools implemented supports aimed at improving student engagement (e.g., live virtual lessons and support, expectations of teachers to provide feedback to students, and materials and advice to parents/guardians to improve the home learning environment). Unfortunately, it does not appear that these supports were able to overcome the challenges of inequalities in technology use and access. Schools, with little time to prepare for the transition to remote learning, appear to have made significant efforts to support their students, even if their students had difficulty accessing online content.

It was clear that the learning experiences of students globally would be impacted by the pandemic. Schools were forced to close and educational leaders were confronted with several challenges in how they would maintain learning continuity with students not able to attend class in-person. In response, many schools implemented remote learning strategies. Given differences in schools’ preparedness to offer such remote learning options, many anticipated the transition to remote learning to exacerbate existing educational inequalities. Our findings partly support this hypothesis. Existing inequalities in access to and confidence in using technology across and within countries likely significantly altered the learning experience of certain students in a negative way, keeping them less engaged in the learning material. However, school efforts in some countries to prepare teachers to support students in a remote setting might have reduced some of the inequalities that might have been expected. The resilience of schools in responding to the COVID-19 disruption possibly limited some of the negative consequences to students, regardless of their background.

## Data Availability

The datasets supporting the conclusions of this article are openly accessible and can be downloaded as public use files from the IEA’s website: https://www.iea.nl/data-tools/repository/reds.

## Appendix

See Tables 8, 9, 10 and 11.

**Table 8:** Items used for constructing remote learning quality scales

Remote learning quality component	Scale [REDS item]	Item description	Levels
Available and Suitable Technology	Access to quality of technology [IS1G03, IS1G05 A-C]	During the [COVID-19 disruption], did you have access to the internet at home?	1: Yes, it worked well all the time 2: Yes, most of the time 3: Yes, but it did not work very well 4: No
		During the [COVID-19 disruption], which of the following devices did you use at home?	1: Yes and it worked well2: Yes but it did not work well 3: No
	Self-efficacy in technology use [IS1G07 A-I]	Before, during, and after the [COVID-19 disruption], how well could you do each of the following tasks? (e.g., Use applications to communicate with my teacher; Fix problems when my device is not working properly)	0: I learned how to do this during the [COVID-19 disruption] or I still do not know how to do this1: I already knew how to do this before the [COVID-19 disruption]
Effective teachers	Teacher support [IS1G21 A-H]	To what extent do you agree or disagree with the following statements about the support you received from your teachers during the [COVID-19 disruption]? (e.g., My teachers were available when I needed their help)	1: Strongly agree 2: Agree 3: Disagree 4: Strongly disagree
Engaged students	Engagement [IS1G18 A-J]	During the [COVID-19 disruption], did the following aspects of your schoolwork change? (e.g., My motivation to complete my schoolwork)	0: Did not change or decreased1: Increased

Full item wording can be found in the appendix of UNESCO & IEA (2022, p. 119)

**Table 9:** N of complete cases before- and after-imputation

	N	N complete cases	N to-be-imputed	N after-imputation
Effective teachers				
Denmark	1444	1206 (84%)	42 (3%)	1248 (86%)
Ethiopia	1952	1583 (81%)	195 (10%)	1778 (91%)
Kenya	1305	1185 (91%)	44 (3%)	1229 (94%)
Russian Federation	3357	3198 (95%)	73 (2%)	3271 (97%)
Slovenia	2552	2448 (96%)	45 (2%)	2493 (98%)
United Arab Emirates	2697	2473 (92%)	75 (3%)	2548 (94%)
Uzbekistan	2570	2546 (99%)	14 (1%)	2560 (100%)
Self-efficacy				
Denmark	1444	1359 (94%)	22 (2%)	1381 (96%)
Ethiopia	1952	1701 (87%)	200 (10%)	1901 (97%)
Kenya	1305	1219 (93%)	58 (4%)	1277 (98%)
Russian Federation	3357	3199 (95%)	73 (2%)	3272 (97%)
Slovenia	2552	2482 (97%)	31 (1%)	2513 (98%)
United Arab Emirates	2697	2537 (94%)	79 (3%)	2616 (97%)
Uzbekistan	2570	2497 (97%)	35 (1%)	2532 (99%)
Engaged students				
Denmark	1444	1218 (84%)	61 (4%)	1279 (89%)
Ethiopia	1952	1575 (81%)	206 (11%)	1781 (91%)
Kenya	1305	1141 (87%)	90 (7%)	1231 (94%)
Russian Federation	3357	3198 (95%)	73 (2%)	3271 (97%)
Slovenia	2552	2430 (95%)	61 (2%)	2491 (98%)
United Arab Emirates	2697	2446 (91%)	104 (4%)	2550 (95%)
Uzbekistan	2570	2533 (99%)	23 (1%)	2556 (99%)

N corresponds to the filtered sample explained in Table 1. Complete cases corresponds to the cases that do not have missing items in that scale. To-be-imputed corresponds to incomplete cases that were imputed a value (i.e., presenting valid response in at least half of the items in that scale). After-imputation corresponds to the complete cases after the values have been imputed. Percentages within parentheses are relative to filtered sample and are unweighted

**Table 10:** Reliability and measurement invariance of scales

	Effective teachers	Self-efficacy in technology use	Engaged students
Cronbach’s Alpha			
Denmark	0.91	0.84	0.89
Ethiopia	0.92	0.92	0.94
Kenya	0.92	0.89	0.92
Russian Federation	0.92	0.88	0.89
Slovenia	0.91	0.87	0.91
United Arab Emirates	0.94	0.90	0.90
Uzbekistan	0.89	0.81	0.88
Measurement invariance models fit			
Configural invariance			
CFI	0.99	0.97	1.00
RMSEA	0.08	0.07	0.03
TLI	0.99	0.95	0.99
Metric invariance			
CFI	0.99	0.96	0.98
RMSEA	0.08	0.07	0.05
TLI	0.99	0.95	0.98
Scalar invariance			
CFI	0.98	0.93	0.99
RMSEA	0.10	0.09	0.04
TLI	0.99	0.92	0.99

**Table 11:** Items used for school-provided supports/resources for remote learning

Support for:	School support/ resource [REDS Item]	Item wording	Levels	School support indicator coding (yes/no)
Access to quality technology	Providing internet/digital devices to students [IP1G03B,D]	Did your school provide any of the following resources during the [COVID-19 disruption]? [e.g., internet, digital devices]	1 = Yes, this was already provided before the [COVID-19 disruption] 2 = Yes, this was provided during the [COVID-19 disruption] 3 = No	Yes = Respond 1 or 2 to both items No = otherwise
	Distributing paper materials or using radio/TV broadcasts [IP1G04C,I]	During the [COVID-19 disruption], did your school make the following resources available to students to support their learning?[e.g., paper materials, radio/TV broadcasts]	1 = Yes, to all students 2 = Yes, to some students 3 = No	For Ethiopia and Kenya: Yes = Respond 1 or 2 to at least one item No = otherwise
Effective teachers	Increasing the use of resources to support remote teaching [IP1G12A-E]	During the [COVID-19 disruption], how did the use of the following resources and activities change in comparison with the time before the [COVID-19 disruption]?	1 = Substantially increased 2 = Increased to some degree 3 = Did not change 4 = Decreased to some degree 5 = Substantially decreased	Coded items separately Yes = 1 or 2 No = 3, 4, or 5
Engaged learners	Teachers providing live support for students [IP1G04D-F]	During the [COVID-19 disruption], did your school make the following resources available to students to support their learning?[e.g., live virtual lessons, live virtual teaching support]	1 = Yes, to all students 2 = Yes, to some students 3 = No	Coded items separately Yes = 1 or 2 No = 3
	Teachers expected to provide feedback [IP1G10A-E]	During the [COVID-19 disruption], were teachers in your school expected to provide feedback to students in the following ways? (e.g., through virtual meetings, emails, phone calls).	1 = Expected and required 2 = Expected but not required 3 = Not expected	Yes = Respond 1 or 2 to at least one item No = otherwise
	Providing resources to parents/guardians to improve home support [IP1G16C,D]	During the [COVID-19 disruption], did your school provide any of the following support for [parents/guardians]? (e.g., teaching material worksheets, advice on how to help child)	1 = Yes, this was already provided before the [COVID-19 disruption] 2 = Yes, this was provided during the [COVID-19 disruption] 3 = No	Coded items separately Yes = 1 or 2 No = 3

Full item wording can be found in the appendix of UNESCO & IEA (2022, p.51)

## Footnotes

1 We also acknowledge the vital role that self-regulation skills play in keeping students engaged and motivated to learn while outside the physical classroom without the supervision of the teacher (Fredricks et al., 2004). Furthermore, learning routines taught in the classroom likely supported students in continuing their learning during asynchronous sections (Kauffman, 2015).

2 Schools were the first sampling stage and students and teachers were the second sampling stage.

3 More details on the REDS sampling design and methodology can be found in Meyer et al. (2022).

4 This time period is also referred to as the “reference period” or “disruption period” in Meinck et al. (2022). We use these terms interchangeably throughout this paper.

5 The option for “Other” in the gender question was administered in only two countries in our sample (Denmark and Kenya). In these two countries, only a small proportion of students chose this category with the highest being 4% in Denmark. Because of this, we do not report findings for this group in the results.

6 In regards to gender differences, it is important to note that results from the International Computer and Information Literacy Study (ICILS) 2013 show that girls tend to report lower levels of self-efficacy for advanced or specialized ICT tasks, even when girls outperform boys in the computer and information literacy test (Fraillon et al., 2014). In contrast, REDS only asked students about basic ICT-related tasks, not advanced or specialized tasks. ICILS 2013 did not find a significant gender gap when examining basic ICT self-efficacy measures; however, in our analysis, we do find significant gender differences in multiple countries.
